# High rate of epidermal growth factor receptor-mutated primary lung cancer in patients with primary breast cancer

**DOI:** 10.3389/fonc.2022.985734

**Published:** 2022-10-13

**Authors:** Tianyu Zeng, Hai Xu, Yincheng Liu, Chunxiao Sun, Fan Yang, Yan Liang, Xiang Huang, Ziyi Fu, Wei Li, Yongmei Yin

**Affiliations:** ^1^ Department of Oncology, the First Affiliated Hospital of Nanjing Medical University, Nanjing, China; ^2^ Department of Radiology, the First Affiliated Hospital of Nanjing Medical University, Nanjing, China; ^3^ Department of Plastic Surgery, the First Affiliated Hospital of Nanjing Medical University, Nanjing, China; ^4^ Nanjing Maternal and Child Health Medical Institute, Obstetrics and Gynecology Hospital Affiliated of Nanjing Medical University, Nanjing, China

**Keywords:** neoplasms, multiple primary, breast neoplasms, lung neoplasms, epidermal growth factor receptor

## Abstract

**Background:**

With increased survival in breast cancer, resulting from advances in treatment, patients incur the possibility of subsequent primary malignancies, especially lung cancer. The aim of this study was to assess the frequency of CT-detected pulmonary ground-glass nodules and lung cancer following breast cancer diagnosis, the associations between breast cancer and lung cancer, the pathological features of double primary cancer, and the status of epidermal growth factor receptor (EGFR) mutations in second primary lung cancer.

**Methods:**

Clinical data from more than 9000 individuals who were diagnosed with primary breast cancer at Jiangsu Province Hospital (Jiangsu, China) between January 2008 and December 2021 were retrospectively analyzed.

**Results:**

Of the 9179 patients, 6512 underwent diagnostic CT, 55 (0.8%) were diagnosed with a second primary lung cancer, which accounted for approximately 18.4% of the pulmonary ground-glass nodules (GGNs) detected. The incidence was higher than in the general female population (standardized incidence ratio 1.4 [95% confidence interval (CI): 1.25-1.55]). Patients who experienced a second primary lung cancer exhibited a significantly higher rate of EGFR mutation (78.5%) than those with lung adenocarcinoma alone, with most exhibiting low-grade malignancy, older age, estrogen receptor negativity, low Ki67, and no lymph node metastasis.

**Conclusions:**

Breast cancer patients, especially those with low-grade malignancy, were at high risk for developing primary lung cancer. For isolated GGN in patients with high-risk factors, clinicians should insist on close follow-up. Furthermore, EGFR may play an important role in primary lung adenocarcinomas and breast cancer.

## Introduction

Breast cancer and lung cancer are the two most frequently diagnosed malignancies, have the highest morbidity, and are the leading causes of cancer-related deaths in women worldwide (1). Advances in treatment and improved surgical techniques have extended the lifespan of breast cancer patients. However, with this prolonged survival, the risk of a second primary cancer has also increased. Approximately 10% of breast cancer patients experience another malignancy within 10 years after diagnosis, 5% of which are lung cancers (2, 3).

What also warrants attention is that the number of women with a double primary cancer may also be increased. The most frequently observed second primary cancers in breast cancer patients are hematological tumors, melanomas, and cancers of the digestive tract, female reproductive system, lung, thyroid, ovary, and urinary tract (4–8). Many studies have demonstrated that a second primary cancer is associated with a lower survival rate (9, 10). However, little is known about secondary lung cancers that follow breast cancer. Although several epidemiological studies have investigated lung cancer secondary to breast cancer (11–15), assessed high-risk factors (16–19) for double primary malignancies, and examined the directional associations of estrogen receptor (ER) status (16) in second lung cancers, the clinical characteristics of primary lung cancer following breast cancer have not been comprehensively described. Therefore, this study aimed to evaluate breast cancer patients with a second primary lung cancer to explore the clinicopathological characteristics of primary lung cancer after breast cancer and to analyze the related associations of breast cancer with primary lung cancer, especially the mutation of EGFR in lung cancer, which may provide new insights for more precise treatment of tumors.

## Materials and methods

### Study population

A review of medical charts at the Jiangsu Province Hospital (Jiangsu, China) revealed 9179 patients with pathologically confirmed breast cancer between January 2008 and December 2021. During this period, 6512 consecutive patients underwent diagnostic chest computed tomography (CT), and ground-glass nodules (GGNs) were detected in 1195. Among them, 403 patients had complete clinical data about clinicopathological characteristics. Retrospective analysis of these patients’ medical records revealed a total of 55 patients with histologically confirmed lung cancer. Clinical information, including pathology and immunohistochemistry of cancer tissues, EGFR status, date of GGN detection, and cancer stage, was collected. All the CT images were evaluated by two senior radiologists retrospectively.

### Follow-up

All patients were evaluated at 3- or 4-month intervals by chest CT. CT findings, including lesion size and radiological features, were confirmed by at least two radiologists. Imaging characteristics were visually classified into four subgroups: pure GGN (pGGN), mixed GGN (mGGN), subsolid GGN (ssGGN), and solid GGN (sGGN).

### Statistical analysis

Statistical analysis was performed using SPSS version 21.0 (IBM Corporation, Armonk, NY, USA). Measurement data are expressed as mean ± standard deviation, and the t-test was used for comparison between groups. The chi-squared test was used for categorical variables, and Fisher’s exact test was used for data in which more than 20% of cells had expected frequencies < 5. The correlation of breast cancer factors with primary lung cancer was analyzed using logistic regression analysis. The standardized incidence ratio (SIR) of lung cancer in patients with breast cancer was then calculated by dividing the number of observed cases by the number of expected cases in the general Chinese population. P-values < 0.05 were considered to be statistically significant.

The study was approved by the Ethics Committee of the First Affiliated Hospital of Nanjing Medical University. Clinical data were collected from patients after obtaining informed consent.

## Results

### The incidence of primary lung cancer in patients with primary breast cancer

A total of 9179 patients with breast cancer were eligible for analysis. Considering that only 6512 underwent diagnostic CT, GGNs were detected in 1195 (18.4%), and a definitive diagnosis of lung cancer was made in 55 (0.8%). Based on the incidence of cancer in China in 2015 (20), higher rates of second primary lung cancer occurred among breast cancer patients compared with the general female population (SIR 1.4 [95% CI 1.25–1.55]).

### Clinicopathological characteristics of patients with double primary cancers

The clinicopathological characteristics of patients with breast cancer, including age, family history of malignancy, tumor size, histological type, lymph node metastasis, and clinical grade, are summarized in **Table 1**. Patients who experienced a second primary lung cancer were more likely to be older (p = 0.04). Patients with ER-negative breast cancer (p = 0.01), low Ki67 (p = 0.01), and no lymph node metastasis (p = 0.01) were more likely to develop a second primary lung cancer. There were no statistically significant differences in the other characteristics.

**Table 1:** Clinicopathological characteristics of breast cancer patients.

Variable	BC-LC	BC-GGN	p-value	SIR
Age, years			0.0407	
≤ 50	22	204		0.45
> 50	33	144		2.19
Family history of malignancy			0.2439	
Yes	16	248		2.46
No	38	94		1.47
Unknown	1	6		0.08
Tumor size			0.2430	
≤ 2 cm	36	85		1.19
>2 cm	19	263		2.77
Estrogen receptor			0.0101	
Positive	42	171		1.03
Negative	13	177		2.11
Progesterone receptor			0.0133	
Positive	29	157		1.08
Negative	26	191		1.04
HER2			0.1697	
Positive	19	105		1.32
Negative	36	243		0.93
Ki67			0.0109	
<30%	33	114		1.79
≥30%	17	152		1.02
Unknown	5	82		0.95
Lymph node metastasis			0.0136	
Yes	11	169		1.07
No	44	179		2.19
Grade			0.1799	
1-2	41	245		3.04
3	14	103		0.93
History of smoking			0.4633	
Yes	2	18		1.05
No/Unknown	53	330		0.99
Radiotherapy			0.7714	
Yes	31	186		0.99
None/Unknown	24	162		1.16

Data are presented as n unless otherwise indicated.

BC, breast cancer; HER2, human epidermal growth factor receptor 2; LC, lung cancer; SIR, standardized incidence ratio.

**Figure 1:**
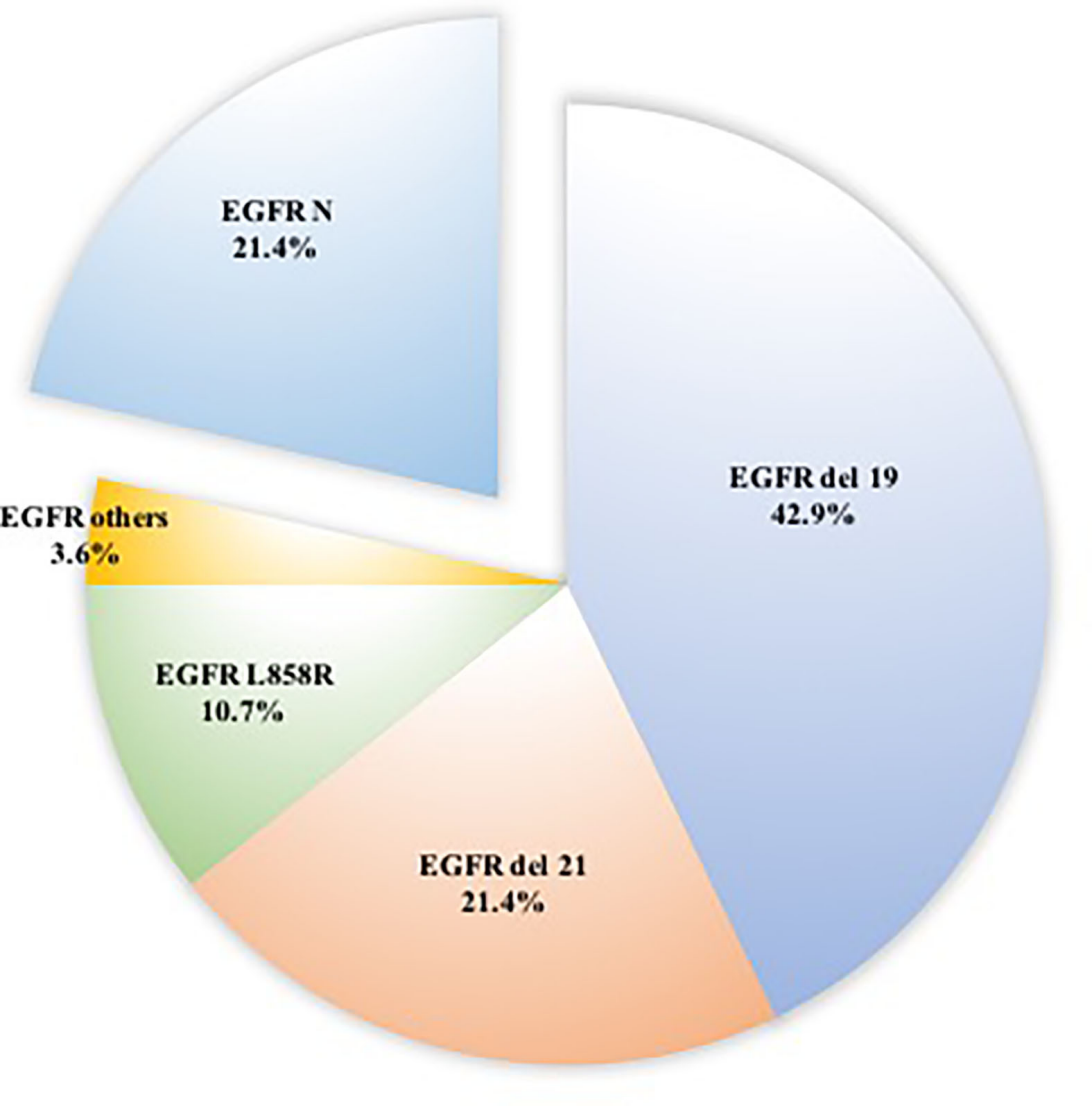
Distributions of 22 patients with EGFR mutation in surgically resected ground-glass nodules.

**Figure 2:**
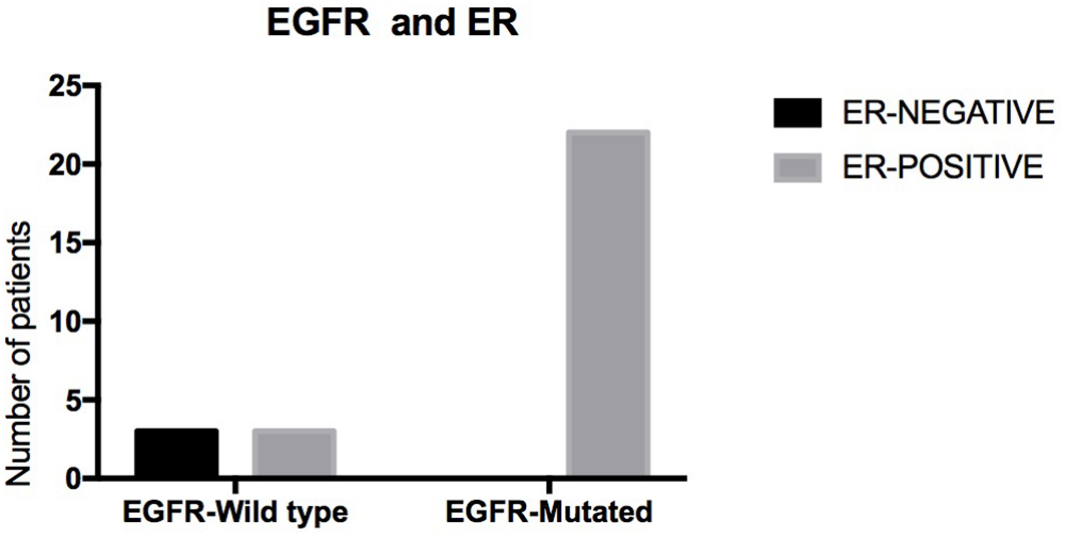
Relevance of status of EGFR and ER.

Of the 55 patients, 54 (98.2%) had lung adenocarcinoma and 1 (1.8%) had small cell lung cancer. Among these patients, EGFR status in lung tissue was assessed in 28, with an EGFR mutation rate of 78.5% (n = 22) (**Figure 1**). We analyzed the relationship between EGFR mutation and breast cancer subtype and found that ER-positive patients were more likely to have EGFR mutations (**Table 2A**, **Figure 2**). The progesterone receptor, human epidermal growth factor 2 (HER2), and Ki67 status had no statistically significant correlation with EGFR status (**Tables 2B**–**D**).

**Table 2A:** Correlation of EGFR mutation of lung cancer and ER status of breast cancer.

No.		ER		Total
		Negative	Positive	
EGFR	Wild-type	3	3	6
	Mutated	0	22	22
Total		3	25	28

Fisher’s exact test, p = 0.006.

EGFR, epidermal growth factor receptor; ER, estrogen receptor.

**Table 2B:** Correlation of EGFR mutation and PR status of breast cancer.

No.		PR		Total
		Negative	Positive	
EGFR	Wild-type	4	2	6
	Mutated	7	15	22
Total		11	17	28

Fisher’s exact test, p = 0.174.

EGFR, epidermal growth factor receptor; PR, progesterone receptor.

**Table 2C:** Correlation of EGFR mutation and HER2 status of breast cancer.

No.		HER2		Total
		Negative	Positive	
EGFR	Wild-type	3	3	6
	Mutated	7	15	22
Total		10	18	28

Fisher’s exact test, p = 0.634.

EGFR, epidermal growth factor receptor; HER2, human epidermal growth factor receptor 2.

**Table 2D:** Correlation of EGFR mutation and Ki67 expression of breast cancer.

No.		Ki67		Total
		<30%	≥30%	
EGFR	Wild-type	4	2	6
	Mutated	13	9	22
Total		17	11	28

Fisher’s exact test, p = 0.73.

EGFR, epidermal growth factor receptor.

We performed immunohistochemistry on 20 lung tumors. All patients were diagnosed as ER-negative, 95% (n = 19) had wild-type anaplastic lymphoma kinase (ALK), and 85% (n = 17) exhibited low Ki67 (< 30%).

### CT imaging

Of the 55 patients with primary breast cancer and lung cancer who underwent CT, pGGNs were reported in 54.5% (n = 30), whereas mGGNs were found in 32.7% (n = 18), sGGNs in 10.9% (n = 6), and ssGGN in 1.8% (n = 1). The average tumor size among all patients was 14.2 mm (range, 4–29 mm).

All patients with wild type EGFR (n = 6) exhibited pGGNs. Among patients with EGFR mutations, 7 (31.8%) had pGGNs, 10 (45.5%) had mGGNs, and 5 (22.7%) had sGGNs (**Table 3A**; p = 0.012).

**Table 3A:** Correlation of EGFR and type of GGN.

No.		GGN			Total
		pGGN	mGGN	sGGN
EGFR	Wild-type	6	0	0	6
	Mutated	7	10	5	22
Total		13	10	5	28

Fisher’s exact test, p = 0.012.

EGFR, epidermal growth factor receptor; GGN, ground glass nodules; pGGN, pure GGN; mGGN, mixed GGN; sGGN, solid GGN.

At the first follow-up CT, the majority of patients (63% [n = 34]) exhibited no changes in GGN size. GGN regression at the first follow-up was observed in 1 patient who underwent endocrine therapy. Thirteen patients (46.4%) with EGFR mutation exhibited stable lesions at the first follow-up (**Table 3A**), but without statistical difference (**Table 3B**; p = 0.136).

**Table 3B:** Correlation of EGFR and change in CT imaging of first time to follow up.

No.		Change in CT		Total
		Stable lesion	Lesion progression
EGFR	Wild-type	6	0	6
	Mutated	13	9	22
Total		19	9	28

Fisher’s exact test, p = 0.136.

EGFR, epidermal growth factor receptor; CT, computed tomography.

### Sites and intervals of double primary cancer

Upon initial review of the 55 patients with double primary malignancy, 22 (40%) exhibited tumors on the same side, 32 (58.2%) had contralateral cancer (lung cancer occurring on the side opposite to the breast cancer), and 1 (1.8%) exhibited bilateral breast cancer. As shown in **Table 4**, the chi-square value was 3.422 (P = 0.064)

**Table 4:** Site of spontaneous breast and lung cancer lesions.

No.		Lung cancer		Total
		Left	Right
Breast cancer	Left	9	20	29
	Right	14	11	29
Total		23	31	54

Chi-square 3.42 p = 0.064.

The interval from breast cancer surgery to diagnosis of lung cancer ranged from 0–420 months, with a mean of 35 ± 13.0 months. Notably, 50 patients were first diagnosed with breast cancer, 3 had breast cancer and lung cancer discovered at the same time, and only 2 developed lung cancer before breast cancer.

## Discussion

Metastasis of breast cancer to the lungs is relatively common in clinical practice (21). Therefore, in breast cancer patients who exhibit pulmonary nodules, lung metastasis is often the first diagnosis considered. However, a certain number of patients with breast cancer and primary lung cancer experience complications of solitary pulmonary nodules. Therefore, it is necessary to understand the frequency of primary lung cancer in breast cancer patients.

To address the deficiency of information regarding the development of a second primary cancer following breast cancer, we retrospectively reviewed data from the Jiangsu Province Hospital to analyze all patients with breast cancer and second primary lung cancer, as well as those with breast cancer diagnosed as a second primary cancer after lung cancer, between 2008 and 2021. Our research provides further evidence demonstrating that the occurrence of lung cancer is closely related to the development of breast cancer, especially lung adenocarcinoma with EGFR mutation. As breast cancer patients live longer, there is an increased possibility of developing subsequent primary lung cancer owing to underlying genetic or other factors (22). An interesting phenomenon in our study was the higher frequency of second lung cancer in patients with low-grade malignancies, which differed from our expectations. Breast cancer patients who were older, had ER-negative cancer, had a low Ki67 index, and displayed no lymph node metastasis exhibited a significantly higher rate of development of a second primary lung cancer. The characteristics of second lung cancer were strikingly similar: 100% were ER-negative, 95% had wild-type ALK, and 85% exhibited low Ki67 (<30%). Further, most patients had stable nodules at the first follow-up. We speculate that low malignancy contributes to longer survival times in cancer patients, and, given that the mean interval of diagnosis of double primary cancers was 35 ± 13.0 months, this was sufficient to permit the development of a second primary lung cancer.

Many previous studies have reported that second primary lung cancer rates are significantly higher in breast cancer patients than in patients with other primary cancers (10, 18, 22), which is consistent with the results of our study. The incidence of secondary primary lung cancer in breast cancer patients may have previously been higher, as there was no pathological diagnosis in the remaining 345 patients with stable GGNs. Chest CT at regular intervals could result in increased detection of pulmonary nodules. Further, the occurrence of stable GGN may be related to common risk factors, including genetics, heredity, hormones, and environmental factors. Previous studies have reported that breast cancer patients who undergo radiotherapy (17, 23), smokers (24), and those treated with chemotherapy (13) have a higher possibility of developing secondary lung cancer.

Nevertheless, the higher risk for developing lung cancer in patients with primary breast cancer cannot be explained merely by regular follow-up. An interesting finding of our study was that the rate of EGFR mutation was as high as 75.6%, which is almost twice that in patients with non-small cell lung cancer without another primary cancer (25). This phenomenon has not been reported in previous studies. These observations suggest that EGFR signaling may play a crucial role in the development of concurrent lung and breast cancers. Several studies have reported that overexpression of EGFR is common in breast cancer patients and is associated with decreased survival (26–29). Moreover, previous reports have indicated that ER signaling plays an important role in primary lung cancer following breast cancer, and that activation of ER signaling occurs through EGFR/HER-1, thus confirming a correlation between ER expression and EGFR mutation (30–35). Patients with an acquired resistance to EGFR antagonists may, therefore, benefit from antiestrogen therapy (14). Moreover, EGFR and HER2 are members of the human epidermal growth factor receptor family, which are type I transmembrane growth factor receptors that activate intracellular signaling pathways and are major determinants of human cancer (36). According to previous studies, overexpression of EGFR is associated with apoptosis, angiogenesis, and formation of tumor vessels. Therefore, EGFR mutation may provide clues of a common etiological pathway between primary lung adenocarcinoma and breast cancer (37, 38).

Patients with a second primary lung cancer were less likely to have pGGNs and more likely to have sGGNs, and those with EGFR mutations exhibited a similar trend (P = 0.05). Analysis of the correlation between CT image patterns and gene mutations demonstrated no statistical significance. Similarly, most of the GGNs had not changed in size at the first follow-up. Unexpectedly, the lesion in one of the patients who underwent endocrine therapy shrunk, consistent with a previous report that breast cancer patients with second primary lung cancers who are treated with antiestrogen therapy exhibit longer cancer-specific survival (14, 34).

There were several limitations to the present study, the first of which was its retrospective, as opposed to prospective, design. Second, random variation and low statistical power resulted from the limited number of patients. Finally, the lack of long-term follow-up prevented us from observing more cancer-related events and assessing cancer-specific survival. The next step is to further follow up the patient, collect more clinical samples, and conduct basic research to explore its underlying mechanisms.

## Conclusions

We observed that women diagnosed with breast cancer demonstrated an increased risk of second primary lung cancer. The present study is the first to report a higher rate of EGFR mutations in second primary lung cancer, which may play an important role in the development of double primary breast and lung cancer. This is an interesting clinical finding that can further the exploration of the mechanism behind elevated EGFR expression in patients with primary breast cancer and the mechanism of EGFR expression in lung cancer and breast cancer, paving the way for development of new drugs. Based on the results of the current research, we recommend that breast cancer patients who exhibit high-risk factors be closely followed. EGFR-targeted treatment represents an alternative option for these patients.

## Data availability statement

The original contributions presented in the study are included in the article/supplementary material. Further inquiries can be directed to the corresponding authors.

## Author Contributions

TZ: Conceptualization, data curation, formal analysis, software, writing-original draft, and writing-review and editing. HX: Conceptualization, data curation, formal analysis, writing-original draft, and writing-review and editing. YiL: Conceptualization, data curation, formal analysis, writing-original draft, and writing-review and editing. CS: Data curation, formal analysis, and writing-review and editing. FY: Data curation, and writing-review and editing. YaL: Data curation and writing-review and editing. XH: Formal analysis and writing-review and editing. ZF: Formal analysis and writing-review and editing. WL: Conceptualization, formal analysis, and writing-review and editing. YY: Conceptualization, formal analysis, funding acquisition and writing-review and editing. All authors contributed to the article and approved the submitted version.

## Funding

This study was financially supported by the National Key Research and Development Program of China (ZDZX2017ZL-01), High-level innovation team of Nanjing Medical University (JX102GSP201727), Wu Jieping Foundation (320.6750.17006), Key medical talents (ZDRCA2016023), 333 Project of Jiangsu Province (BRA2017534 and BRA2015470), The collaborative innovation center for tumor individualization focuses on open topics (JX21817902/008), Postgraduate Research&Practice Innovation Program of Jiangsu Province (SJCX21_0625) and Project of China key research and development program precision medicine research (2016YFC0905901).

## Acknowledgments

The authors would like to thank Lei Wang for excellent technical support and Professor Xichun Hu for critically reviewing the manuscript.

## Conflict of interest

The authors declare that the research was conducted in the absence of any commercial or financial relationships that could be construed as a potential conflict of interest.

## Publisher’s note

All claims expressed in this article are solely those of the authors and do not necessarily represent those of their affiliated organizations, or those of the publisher, the editors and the reviewers. Any product that may be evaluated in this article, or claim that may be made by its manufacturer, is not guaranteed or endorsed by the publisher.
